# Describing the Situational Contexts of Sweetened Product Consumption in a Middle Eastern Canadian Community: Application of a Mixed Method Design

**DOI:** 10.1371/journal.pone.0044738

**Published:** 2012-09-21

**Authors:** Jean-Claude Moubarac, Margaret Cargo, Olivier Receveur, Mark Daniel

**Affiliations:** 1 École de Santé Publique, Université de Montréal, Montréal, Canada; 2 School of Health Sciences, University of South Australia, Adelaide, Australia; 3 Département de Nutrition, Université de Montréal, Montréal, Canada; The University of Hong Kong, Hong Kong

## Abstract

Little is known about the situational contexts in which individuals consume processed sources of dietary sugars. This study aimed to describe the situational contexts associated with the consumption of sweetened food and drink products in a Catholic Middle Eastern Canadian community. A two-stage exploratory sequential mixed-method design was employed with a rationale of triangulation. In stage 1 (n = 62), items and themes describing the situational contexts of sweetened food and drink product consumption were identified from semi-structured interviews and were used to develop the content for the Situational Context Instrument for Sweetened Product Consumption (SCISPC). Face validity, readability and cultural relevance of the instrument were assessed. In stage 2 (n = 192), a cross-sectional study was conducted and exploratory factor analysis was used to examine the structure of themes that emerged from the qualitative analysis as a means of furthering construct validation. The SCISPC reliability and predictive validity on the daily consumption of sweetened products were also assessed. In stage 1, six themes and 40-items describing the situational contexts of sweetened product consumption emerged from the qualitative analysis and were used to construct the first draft of the SCISPC. In stage 2, factor analysis enabled the clarification and/or expansion of the instrument's initial thematic structure. The revised SCISPC has seven factors and 31 items describing the situational contexts of sweetened product consumption. Initial validation of the instrument indicated it has excellent internal consistency and adequate test-retest reliability. Two factors of the SCISPC had predictive validity for the daily consumption of total sugar from sweetened products (Snacking and Energy demands) while the other factors (Socialization, Indulgence, Constraints, Visual Stimuli and Emotional needs) were rather associated to occasional consumption of these products.

## Introduction

Independent public health authorities acknowledge that the global production and high consumption of processed foods and drinks are major factors implicated in the current epidemic of obesity and related chronic diseases [1]–[3]. To address this issue, Brazilian researchers introduced a novel classification of foodstuffs based on the nature, extent and purpose of food processing [4]. The classification divides all foodstuffs into three groups: unprocessed or minimally processed foods; processed culinary ingredients; and ultra-processed products. High consumption of ultra-processed products is proposed to be the most significant problem in the context of obesity and chronic diseases [5]–[7].

The Food and Agriculture Organisation and World Health Organisation (FAO/WHO) Scientific Update Committee [8], [9] as well as the American Heart Association [9] have recommended that individuals limit their daily consumption of processed sources of dietary sugars in order to prevent the onset of chronic diseases. Most of these dietary sugars are found in sweetened food and drink products, a subgroup of ultra-processed products. These products include chocolate, candies, ice creams, sugary baked goods (cookies, cakes, muffins, and other pastries), soft drinks, sweetened juices and beverages, as well as jams and jellies [4]. Some processed culinary ingredients are also high in dietary sugars, including honey, syrups, and sweeteners, use to augment the palatability of beverage and dishes [4].

Sweetened products share several dietary characteristics that may favor weight gain; they are energy dense (for solids) and have a high content in free sugars and often in fats and in saturated fats, while being low in fibre, protein, vitamins and minerals. Sweetened products having a lower satiety effect than protein and fibre rich food, and being rapidly absorbed in the small intestine [6]. Most of these foods are also high in fructose syrup which, when metabolized, may be linked to the development of chronic diseases [10].

Furthermore, in western countries, sweetened products are often sold in large portion sizes and are commonly consumed as snacks both of which may contribute to energy imbalance [11]. Additionally, these products are convenient and attractive, and have a pleasant sweet taste in addition to unique hedonic and psychosomatic properties [12]. In western urban settings, sweetened products are widely accessible in food and non-food venues [13], [14], sold at affordable prices and promoted by effective social marketing strategies [15].

Understanding the situational contexts related to the consumption of sweetened food and drink products may provide insight for prevention-oriented public health policies and specific intervention programs, including social marketing strategies and educational programs aimed at reducing consumption. Research conducted thus far on contextual or situation-based factors that affect food consumption has identified factors including ambience [16], eating location [17]–[19], television viewing [20], family meals [21], meal occasion [22], the presence of others [23], and the price and accessibility of food [24]. However, most such research has been limited to examining just one single situational factor.

Studies examining sweetened product consumption per se have also generally been limited to a focus on a specific type of product such as soft drinks [25], [26] or chocolate [27]–[29]. Instruments and models intended to measure the determinants of food consumption have been developed either for general food consumption [30]–[32] or focused on one dimension of food behaviour such as emotional eating [33], [34] or food craving [35], [36].

To our knowledge no study thus far has simultaneously examined the multiple situational contexts of sweetened product consumption. Such knowledge would be important to orient dietary guidelines especially for urban migrant populations for which exposure to a Western diet rich in sweetened food and drink products has been linked to a greater risk of diabetes and weight gain [37]. In Canada, Arab or Middle Eastern migrants comprise one of the largest non-European ethnic groups and have one of the highest prevalence of overweight/obesity [38]. Middle Easterners are known for the daily consumption of sweetened tea [39]. However, other sweetened products have secondary role in traditional Middle Eastern cuisine in that they are mostly home-prepared pastries and deserts most frequently consumed during festivities and on special occasions [40], [41]. However, Arabs are exposed to a new food environment when they migrate to Canada where sweetened products are abundant, cheap, convenient, accessible, and part of the mainstream food culture. Understanding the situational contexts in which Arab Canadians consume sweetened products would help to orient public health programs aimed at alleviating the problems of obesity in this population.

As a basis for such research, this study sought to identify and describe the situational contexts associated with the daily and occasional consumption of sweetened food and drink products in a Canadian urban migrant community. To fulfill this objective, a two-stage exploratory sequential design was used to develop and assess the initial validation of the self-report Situational Context Instrument for Sweetened Product Consumption (SCISPC).

## General Methods

### Research Design

**Figure 1:**
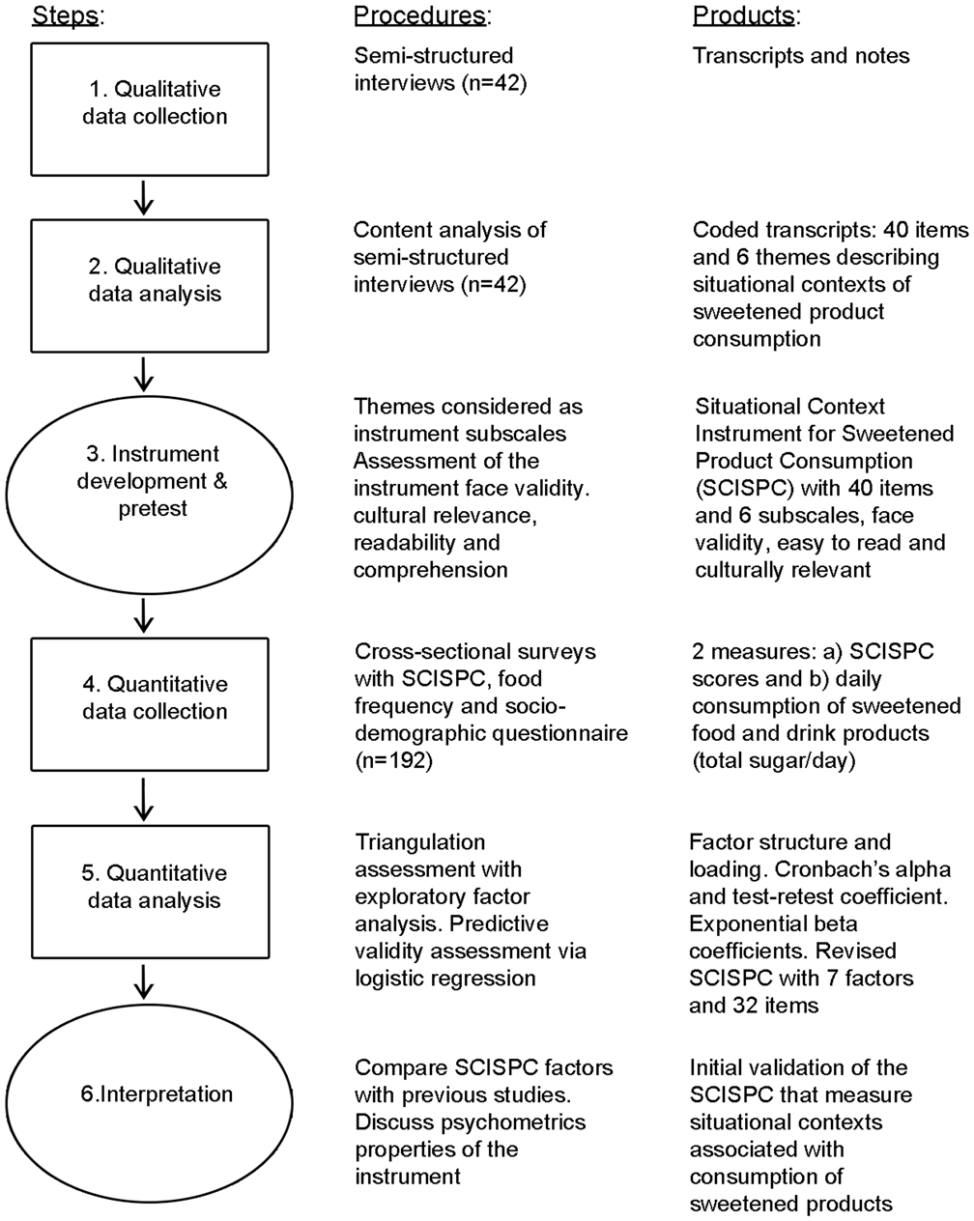
The exploratory sequential design used to develop the Situational Context Instrument for Sweetened Product Consumption in the Catholic Middle Eastern Canadian community.

Mixed-methods designs are recommended for investigating the environmental dimensions of dietary and behavioural practices [42], [43]. This study employed a two-stage exploratory sequential mixed-method design as illustrated in Figure 1 [44] to collect and analyze qualitative and quantitative data. This design enabled a concurrent mixed-method analysis, with a rationale of triangulation where findings from the quantitative data were compared with qualitative results [45]–[48]. The first stage collected and analysed qualitative interview data to identify items and themes that describe the situational contexts of sweetened product consumption. The results of these analyses informed the content for the self-report Situational Context Instrument for Sweetened Product Consumption (SCISPC). The second stage employed exploratory factor analysis to examine the thematic structure emerging from the qualitative analysis as a means of furthering the construct validation of the SCISPC [46]–[48]. The instrument reliability and predictive validity in relation to the total sugars consumed from sweetened food and drink products per day were also assessed at the second stage.

### Target population

The Middle Eastern or Arab community living in Canada is heterogeneous in terms of its country of birth and religious affiliation. The main groups are Lebanese (41%), Egyptian (12%), Syrian (6%), Moroccan (6%), and Iraqi (6%) [49]. Canadians of Arab origin are equally divided between Muslims and Christians, of which the majority is Catholic [49]. Religious affiliation within the Middle Eastern community is essential to acknowledge, especially because religious beliefs impose dietary restrictions amongst Muslims [50], [51].

This study was conducted in an established Catholic Middle Eastern community living in Montreal, Canada. Community members were first and second generation migrants from Egypt, Lebanon and Syria who came to Canada from 1960 onwards. Recruitment and data collection occurred at three Catholic Middle Eastern churches located in Montreal. Participation was solicited through public announcements and was limited to one respondent per household to avoid bias related to family habits. Subjects were all volunteers and could withdraw from the study at any point.

### Ethics Statement

The research protocol was submitted and approved by the ethics committee of the Centre Hospitalier de l'Université de Montréal (SL 06-063). All participants provided their informed, written consent.

### Methods of Stage 1

The first stage (steps 1 to 3 in Figure 1) was exploratory and had two objectives: 1) to develop a self-report instrument to measure the situational contexts associated with the consumption of sweetened products; and 2) to assess the instrument's face validity, cultural relevance, readability and comprehension.

### Design

A fundamental qualitative descriptive design [52] was utilised to develop the content for the Situational Context Instrument for Sweetened Product Consumption. This design provides for a rich and straight description of an experience or event with researchers staying close to the surface of their data in interpreting the meaning of words, experiences and events. We applied a conceptual framework inspired by the Behaviors of Eating and Activity for Children's Health Evaluation System (BEACHES) which was shown to be valid and appropriate for studying influences on diet and physical activity in children in a variety of settings [53]. BEACHES was developed to code direct observations of children's dietary and physical activity behaviors, as well as associated environmental events, including physical location and antecedents. In this study, we adapted several of the BEACHES coding categories that pertained to the environmental events and defined the situational context as the conditions (time, place) and circumstances (surroundings, antecedents) in which the act of consuming sweetened products occurs. Time was defined as the moment of the day when the food or drink product is consumed. Place consisted of the physical location where consumption take place. Surroundings related to the presence or absence of people with which the sweetened product is consumed (friends, family, co-workers or alone). Antecedents were defined as the elements preceding and/or provoking the act of consumption, such as a feeling (e.g., hunger, sadness), an activity (e.g., watching television) or an external stimuli (e.g., sight, offering). Semi-structured interviews were used to collect data on experiences and events related to situational contexts associated with sweetened product consumption.

### Participants

A convenience sample of 42 normoglycemic individuals (self-reporting neither prediabetes nor diabetes per se) (25 women, 17 men) evenly distributed across age groups (18–30 years old; 31–45 years old; 46–60 years old) was employed to develop the SCISPC content. A second sample (n = 20) was recruited for instrument pre-testing.

### Interview procedure

Questions in the semi-structured interview guide were based on the adapted BEACHES conceptual framework and informed by a literature review. Interviews were conducted in a location convenient to participants (i.e., their house, church, or community center). Interviewees were instructed to think about their consumption of sweetened food and drink products; examples were provided. First, participants were queried about the three most recent occasions during which they consumed sweetened products and were asked to describe the conditions (time and place) and circumstances (surroundings and antecedents) associated with each occasion. Next, participants were asked to name one favourite sweetened food or drink product and to talk about the various conditions and circumstances in which they usually consume it. Lastly, participants were questioned about their customary consumption of sweetened products in different places (at home, at work/school, at a restaurant, at the shopping mall, etc.). All interviews were conducted by the lead author and recorded. Interviews lasted an average of 30 minutes.

### Content analysis

Interviews were transcribed and reviewed against the recordings to ensure transcription accuracy. Content analysis was performed using open and focused coding procedures [54] and guided by the conceptual framework. Responses to questions were assigned open codes to capture the situational contexts associated with sweetened product consumption. These codes were defined literally with words used by participants to describe their consumption (e.g., “When I feel sad” or “When I am at a birthday party”). As interviews were coded, codes were compared and contrasted; similar items were grouped under a focused code to represent an overarching theme (e.g. Energy or Emotions). Interviews were transcribed and coded iteratively. An item occurrence matrix was created to monitor the recurrence of open codes. Interviews were conducted until saturation was achieved, that is, until no new open codes emerged.

### Pretest assessment

Face validity of the SCISPC was assessed using various criteria applied to mixed methods studies [44], [55]. Credibility was enhanced by the lead author's familiarity with the target population and ability to gain participants' trust. Also, data were collected until saturation was achieved. Documenting coding decisions through memos combined with regular peer debriefing with co-authors contributed to the researcher maintaining an objective stance during data analysis. The first draft of the instrument was administered to 20 participants to check items for cultural relevance, readability and comprehension. Participants were asked to complete the self-report SCISPC questionnaire on their own time, note any comments and indicate an appropriate time for the researcher to follow-up.

## Results of Stage 1

### Situational contexts of sweetened products consumption

Overall, most participants (n = 36/42) described a specific preference for sweet liking, and were very enthusiastic and self-conscious whilst describing the situational contexts of their consumption. The remaining six participants expressed a preference for salty food, sometimes describing themselves as a ‘*salt person*’. Nevertheless, all individuals reported eating or drinking sweetened food and drink products either regularly or occasionally.

Six themes with 40-items emerged from the analysis to describe the situational contexts associated with the consumption of sweetened food and drink products: 1) Energy, 2) Negative Emotions, 3) Positive Emotions, 4) Social Environment, 5) Physical Environment, and 6) Constraints. Table 1 contains quotes from participants to illustrate each theme. Participants described eating sweets when they felt a drop of energy, a craving or while performing a physical or a mental exercise (energy theme). Most acknowledged that the consumption of sweetened products was triggered by negative emotional feelings, either depression related feelings such as sadness, boredom, and loneliness, or stress-related feelings like anxiety (negative emotion theme). Others talked about eating these products in situations associated with pleasure, reward and self-satisfaction (positive emotion theme). Most participants associated the sharing and offering of sweetened products in social situations such as birthdays, when visiting/receiving friends or relatives, or while sharing a meal (social environment theme). Some admitted that external visual stimuli such as sight or smell might have triggered consumption (physical environment theme) like for example, seeing food lying on the table or after seeing commercial/advertisements. Lastly, some participants reported eating sweetened products in situations where they felt hungry, but were limited in their food choices by temporal or spatial factors (constraint theme).

**Table 1:** Quotes from participants to the semi-structured interviews showing them describing the situational contexts of sweetened food and drink product consumption.

*Energy*
Psychologically, sugar gives me energy (M, 33); Sometimes I need energy and I feel like eating sugar (F, 32); If I have a snack it will be something sweet (F, 29). When I feel weak and I have a sugar craving, I will go and get my sugar my body tells me indirectly that I need sugar (M, 52).
I don't know I always feel the need to eat sugar; I always need sugar (F, 29). It's linked to work, the efficiency, to the amount of hours I estimate working, If I think I will work 4 to 5 hours I need to be efficient, I will eat some brownie, some sugar, some and coffee (M, 33).
*Negative Emotions*
Because when I am sad I need some chocolate, it cheers me up (F, 62); When you feel very nervous or stressed, this is when I would be more willing to eat sweets, in those conditions (M, 33).
When we say we eat our emotions. Sometimes its emotional, it's to fill a void (F, 38).
*Positive emotions*
When I eat chocolate at night it's when I worked all week long spending my energy and effort and I didn't receive anything for myself. When this happens, I feel like rewarding myself, like I deserve it (F, 33). It's just for the pleasure of eating it (F, 50).
*Social Environment*
It's quite rare that you go to someone house and they will offer you a fresh fruit, you always need sugar. (F, 36); It's a social thing, we were at the restaurant and someone with us ordered dessert. I was very tempted to have some myself and I did (M, 25); It's always someone you offers you some sweets. I don't go out myself to buy something out of the vending machine (F, 38); Yes, if I am at a party and there is some dessert, I will have some (M, 41).
*Physical Environment*
I saw it in front of me so I felt like eating it (F, 28); If there is some cake or pie lying on the table I will go and have some (M, 29); I mean, if you have candies you will eat it, if you don't, you won't. You don't look for it, but you have to see it to eat it (F, 62); When I buy sweets it's often when I go to the gas station, when I go in to pay, I must buy a kit-kat or whatever, I can't resist (M, 25).
*6. Constraints*
You know, when there is nothing to eat around, I will eat some chocolate (F, 18); Because of the stress and lack of time, I am forced to eat at restaurants and buy things at the convenient store, I don't have to do it, but I do (M, 33). If I am driving home and I know I will be late, I will eat a snack, one or two piece of chocolate (F, 53). I was hungry and I didn't feel like preparing anything, so I ate some sweets, it was right in front of me (F, 32).

F = Female; M = Male.

### Instrument development

The six themes and 40-items from the qualitative analysis served as the basis for constructing the first draft of the SCISPC. The self-report questionnaire included an example list of 26 sweetened food and drink products shown in Table 2 and derived from a 24 h recall analysis conducted in the same community [56]. Participants had to answer the leading statement ‘I tend to eat sweetened food or drink products’ for each of the 40 situational contexts using a five point Likert answer scale: (1) strongly disagree, (2) disagree, (3) neither agree nor disagree, (4) agree and (5) strongly agree.

**Table 2:** Sweetened food and drink products included in the food frequency questionnaire with their total sugar content.

Sweetened products	Total sugar content^1^
Baklava	25–30%
Brownie	26–28%
Bun	20–45%
Cake	24–46%
Candy	42–75%
Chewing gum	100%
Chocolate (bars and spread)	57–99%
Chocolate milk	57–61%
Coffee (sweetened)	36–87%^2^
Cookie	16–33%
Donut	22–26%
Fruits juices and drinks	75–98%
Honey	100%
Ice cream	17–61%
Jam	38–64%
Maple syrup	81%
Muffin	25–30%
Soft drink	84–100%
Sweetened sauces	57–86%
Sweetened yogurt	57–69%
Tea (sweetened)	36–87%^2^

1 Total sugar content refers to the percentage of calories attributed to total sugars in each product. Percentages were calculated using the Canadian Nutrient File and included all varieties of a given product. For example, ice creams products contain between 17% and 61% of calories from total sugars.

2 Considering 1 or 2 tablespoon of sugar.

At pretest, the instrument was judged by community members to be easy to read and to understand, and culturally relevant to adequately capture the situational contexts of sweetened product consumption.

## Methods of Stage 2

A cross-sectional study design was utilised to address the objectives for the second stage (steps 4 to 6 in figure 1) which aimed to: 1) examine the thematic structure emerging from the qualitative analysis using exploratory factor analysis to further the instrument's construct validation; 2) assess the instrument's reliability; and 3) assess the instrument's predictive validity in relation to the daily consumption of sweetened products.

### Participants and instruments

One hundred ninety-two individuals (105 women, 87 men) from the same church communities completed the SCISPC, a food frequency questionnaire, a socio-demographic questionnaire adapted from Statistics Canada [57], and self-reported weight and height. Self-reported normoglycemic status was verified with an HbA1c test using Cholestech GDX instrument [58] and an exclusion cut-off of >6.5% [59].

### Exploratory factor analysis

Exploratory factor analysis was used on the 40 SCISPC items to examine the structure of themes that emerged from the qualitative analysis as a means of furthering construct validation [45], [46]. Data were extracted using the principal component method; factors were rotated by orthogonal transformation using the Varimax rotation. Several recommended guidelines for exploratory factor analysis were used to obtain factor solutions that were adequately stable and corresponded closely to population factors [60], [61]. First, items with communality under 0.60 were dropped to ensure a high level of item communalities; factor solutions were run until this criterion was satisfied for all items and that the mean communality was above 0.70. Second, the number of factors was determined by setting the minimum eigenvalue at 1.0. Third, the factor loading cut-off value was set at 0.50 and items with cross-loading were dropped; the factor solutions were run until no cross-loadings were found. Fourth, the Bartlett's test of sphericity and overall Kaiser-Meyer-Olkin (KMO), as well as KMO for individual items, were used to examine the fit of the data of the final factor solution. Lastly, over-determination in the final factor solution was evaluated using the factor-to-variable ratio, the number of items per factors, and the presence of high loadings items on each factor.

### Reliability

Internal consistency was assessed with Cronbach's alpha calculated for each refined sub-scale of the SCISPC. Test-retest reliability was assessed using the pretest sample of 20 individuals from stage 1; participants completed the questionnaire twice at an interval of two weeks.

### Predictive validity

To identify which situational contexts are predictive of the daily consumption of sweetened food and drink products, SCISPC factor scores were regressed on the dependent measure operationalized as the daily consumption of total sugars from these products using a food frequency questionnaire.

The food frequency questionnaire format included a list of 26 sweetened food and drink products (Table 2). Respondents were asked to report the average number of days per week, in a typical week (i.e., excluding festivities), for which each product was eaten. Participants were also asked to report on how many portions of the product they typically eat or drink. Examples of portions sizes were taken from the Canadian Nutrient Files (CNF) and provided to participants [62]. The daily consumption of sweetened food and drink products was expressed as total sugar and measured as follows:
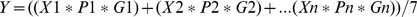

*Y* = Average daily consumption of total sugars

*X*1 = Numbers of days/week of consumption for food item 1

*P*1 = Average eating portions/day for food item 1

*G*1 = Amount of total sugars contain in a mean portion for food item 1

The average amount of total sugars contained in a mean portion of each food item was estimated using CNF. For each item, this amount corresponded to the average amount of total sugars contained in all types of the given item (both commercial and home-made products) available in CNF.

Linear regression was performed using the generalised linear model for non-normally distributed data (gamma distribution and log-link function). Regressions were first performed to verify if sweetened product consumption varied by age and gender. Age and each SCISPC factor score were entered as continuous variables. Statistical validity was assessed using IAC and Pearson square. Beta coefficients were exponentiated to evaluate the magnitude of associations between situational context factors and daily consumption of sweetened products per 1-point interval in the extent of the predictor.

## Results of Stage 2

### Socio-demographic profile

Participants at stage 2 were aged between 18 and 60 years old (Mean = 35; SD = 12.8). Country of birth included Egypt (31.9%), Lebanon (25.1%), Syria (14.7%), and other Middle Eastern countries (5.3%). Individuals migrated between 1962 and 2007, 86% of which arrived before 2000. Another 23.0% of respondents were born in Canada; they were second-generation migrants from parents born in the Middle East region.

The vast majority of respondents had a high school diploma (94%) and most had a university degree (66%). Most participants had a family income above 50K$ (65.9%). No individuals were excluded on the basis of having HbA1c values not consistent with normoglycemic status, but two individuals were excluded from the analysis because of missing answers from the questionnaires.

### Factor structure

Throughout the process, two items were dropped because of a low communality (<0.60). An additional seven items were dropped because of cross-loadings on several factors. The final factor solution had seven factors as follow (with number of items per factor): Emotional needs (8), Snacking (5), Socialization (4), Visual stimuli (4) Constraints (4), Energy demands (4), and Indulgence (2). Factors were moderately correlated (Pearson correlation ranging from 0.31 to 0.64).

Table 3 presents the list of contextual items, each item's initial subscale assignment from stage 1 and final assignment from the factor analysis, in stage 2. The seven factors combined explain 69.4% of the total variance. After rotation, each factor explained between 6.7% and 20.3%. The KMO of the final factor solution was 0.92 and the Bartlett's test of sphericity was significant (P<0.001) which shows an excellent fit of the data and that the sample size was adequate for the analysis. All items retained in the final solution (n = 32) have high communalities (>0.60) with an average communality of 0.70. Eight items originally identified in the content analysis were not retained in the factor structure because of low communalities (including “when I watch television”, “when I am at the movies”, and “when I am convenient store”). The factor-to variable ratio was 4.4. All factors have more than the recommended minimum number of items (3), except for the Indulgence factor. Most factors have several items with high loadings above 0.70.

**Table 3:** Factor structure of the Situational Context Instrument for Sweetened Product Consumption.

Items	Th^1^		Factors^2^						Comm^3^
When I feel nervous	Ne	0.84							0.81
When I am stressed	Co	0.85							0.83
When I feel anxious	Ne	0.84							0.84
When I am angry	Ne	0.84							0.83
When I feel alone	Ne	0.70							0.70
When I feel anguish	Ne	0.83							0.81
When I am sad	Ne	0.80							0.75
When I am bored at home	Ne	0.70							0.74
When I am snacking at work	En		0.70						0.60
While I am at a work break	Pe		0.70						0.65
If I see sweets lying in the table	Pe		0.64						0.61
When I am snacking at home	En		0.66						0.70
If I feel a hungry	En		0.74						0.72
If I am at a special event or birthday	Se			0.62					0.61
When I am with people at a restaurant	Se			0.77					0.70
When I have guest over	Se			0.80					0.69
When I am on a visit	Se			0.72					0.67
When passing in front of a vending machine	Pe				0.68				0.65
When someone is eating in front of me	Se				0.76				0.60
While I am at the gas station	Pe				0.58				0.73
If I see an advertisement on sweets	Pe				0.79				0.71
When I don't have any time to eat	Co					0.70			0.63
When I don't feel like cooking	Co					0.53			0.61
When there is nothing to eat	Co					0.72			0.63
When I can't waste time eating at work	Co					0.60			0.67
If I feel a drop of energy	En						0.75		0.73
If I need a boost of energy	En						0.67		0.76
Before or during physical exercise	En						0.52		0.69
To keep me active working or studying	En						0.68		0.61
To please myself	Po							0.77	0.71
To treat myseflf	Po							0.83	0.77
**Eingenvalues**		6.3	3.5	2.6	2.6	2.2	2.1	2.0	
**Percentage of variance explained**		20.3	11.4	8.4	8.3	7.3	7.0	6.7	

1 Themes to which items were assigned at the end of stage 1: Negative Emotions (Ne), Energy (En), Social Environment (Se). Physical Environment (Pe) and Positive Emotions (Po).

2 Factors are: Emotional needs (1); Snacking (2); Visual Stimuli (3); Constraints (4); Socialization (5); Energy demands (6); Indulgence (7).

3 Communalities for each items.

Overall, the factor solution confirmed the qualitatively derived thematic structure for the SCISPC from stage 1 with minor changes. A new factor named ‘Snacking’ emerged which included some items formerly classified into ‘Energy’ and ‘Physical Environment’. The other domains were retained but were renamed to improve the meaning/interpretation of the thematic label. As illustrated in Table 2, four items were relocated into domains other than the one in which they were originally assigned at stage 1.

### Reliability

Internal consistency analysis for the revised SCISPC was 0.94 with sub-scale alphas as follows: Emotional needs (0.96), Snacking (0.86), Visual stimuli (0.78), Constraints (0.79), Socialization (0.77), Energetic needs (0.78) and Indulgence (0.77). Test-retest reliability of the SCISPC was 0.74.

### Predictive validity

Average daily consumption of sweetened food and drink products was 75.4 grams of total sugars per day (SD = 50,9g/j; n = 190) with a range from 2.9 to 316 g/day. Sweetened product consumption followed a gamma type distribution and was inversely associated with age (Exp.β = 0.98, CI[0.98:0.99], p<0.001). No statistical differences were found by sex.

Table 4 presents the mean scores and standard deviations for each of the seven SCISPC factors (n = 190). Univariate regression analysis results are also shown with exponentiated beta coefficients and p-values. ‘Snacking’ and ‘Energy demands’ were significantly associated with the daily consumption of sweetened food and drink products (p<0.05). The factors ‘Emotional Needs’, ‘Constraints’ ‘Visual Stimuli’, ‘Indulgence’ and ‘Socialization’ were not significantly associated with the daily consumption of sweetened products and corresponded to their occasional consumption.

**Table 4:** Descriptive statistics of the SCISPC and univariate regression of factor scores on sweetened food and drink product consumption (n = 190).

Factors	Descriptive		Regression		Hypothesis		95% Confidence Interval		
	Score^1^	SD	Beta	SE	?	P<	Exp (B)^2^	Low	High
Snacking	3.11	0.98	0.16	0.07	11.04	0.00	1.18	1.07	1.29
Energy demands	3.14	0.92	0.12	0.05	4.71	0.03	1.13	1.01	1.25
Emotional needs	2.66	1.10	0.07	0.04	2.39	0.12	1.08	0.98	1.18
Visual stimuli	2.32	0.86	0.09	0.06	2.41	0.12	1.10	0.98	1.23
Constraints	2.68	0.91	0.10	0.05	3.28	0.07	1.10	0.99	1.23
Socialization	3.44	0.83	−0.04	0.05	0.57	0.45	0.96	0.86	1.08
Indulgence	3.49	1.10	0.05	0.04	0.94	0.33	1.05	0.96	1.14

1 Mean score of all items on a given factor representing the level of agreement on the 5-point Likert scale ranging from 1 (totally agree) to 5 (totally disagree).

2 Per 1-point interval in the extent of the predictor on the 5-point Likert scale.

## Discussion

This study successfully applied a two-stage exploratory sequential design to identify and describe the situational contexts of sweetened food and drink product consumption in a Catholic Middle Eastern Canadian population. To fulfill this objective, a self-report instrument, the Situational Context Instrument for Sweetened Product Consumption, was developed and initially validated within the study community.

Various techniques recommend for mixed methods research were applied in this study, including peer consulting and pretesting with community members to assure that the SCISPC was comprehensible, readable and culturally relevant to adequately capture the situational contexts of sweetened product consumption. Exploratory factor analysis was used to examine the structure of themes that emerged from the qualitative analysis as a means of furthering construct validation of the SCISPC [45], [46]. Using recommended strategies for exploratory factor analysis [60], [61], we were able to show that the final factor solution was characterized by a high level of item communalities without cross loadings, an acceptable level of over-determination of the factors, and an excellent fit of the data. Overall, these criteria ensure that data presented is strong and that the factor structure is stable. Lastly, the SCISPC showed strong psychometric properties, including high internal consistency for overall scale (α = 0.94) and its subscales (α = 0.77–0.96), with adequate test-retest reliability (0.74).

Two of the SCISPC factors had predictive validity for the daily consumption of sweetened products. The first one, ‘Snacking’, indicates that sweetened products are eaten daily as snacks and in situations conducive to snacking; for example, being on a work break, when feeling hungry, or when sweetened foods and drinks are seen in the nearby environment (e.g., laying on the table). This result aligns with the fact that sweetened food and drink products are popular snacks consumed in western countries, including in Canada [63], [64], in US [65] and in Finland [66]. Several intrinsic characteristics of these products may explain why they are prone to snacking; they are highly palatable, cheap, convenient and easily accessible. More importantly, they are specifically designed and branded to encourage snacking [67].

The second factor, ‘Energy demands’, reveals that sweetened products are consumed before or during physical exercise, while performing a mental activity or when one feels a drop in energy. These items relate to a functional value of these products associated with energy. Such a factor was also found in the Attitude to Chocolate Questionnaire (ACQ) with items describing chocolate consumption when exercising or under a feeling of hunger [27]. However we found that energetic demands may also apply to cognitive work. This is supported by a prior study indicating that reading and writing can stimulate food consumption and greater instability of glucose plasma levels up to 45 minutes after the performance of these tasks [68].

Five factors of the SCISPC were not predictive of daily consumption of sweetened products and related only to their occasional consumption. The first one is “Socialization”, which describes social occasions such as birthdays where sweets and desserts are eaten, or when visiting/receiving friends or relatives. A socializing function of several sweetened products has been reported in various settings, such as in shared meals by Californian Mexican families [17] or soft drink consumption amongst Australian teenagers, as a way to participate and socialize with others [25]. In Egypt, home-made pastries and desserts have been traditionally used as festive foods in religious days [40].

The factor ‘Visual stimuli’ suggests that sweetened products are eaten in occasional situations where individuals are stimulated by external visual stimuli, e.g. viewing an advertisement on sweets or passing in front of a vending machine. This interpretation is supported by externality theory, which stipulates that external stimuli, such as sight and smell of foods, can affect the physiological functions which regulate appetite and food intake [69]. Exposure to publicity promoting pleasure and excitement related to high energy food was shown to have a direct effect on sweetened product consumption [70]. The presence of vending machines has also been associated to soft drink and sweetened food consumption in US high-school settings [71].

The third factor, ‘Constraint’, suggests that sweetened products are consumed when individuals are hungry and limited in their food choices by temporal or spatial constraints. In fact, easy-to-obtain products are convenient choices that fit well with the constraints of a busy work schedule or when food choices are limited in the work environment. Several studies reported that work-related time constraints are associated with unhealthy food choices such as eating at fast-food restaurant and consuming less fruits and vegetables [72]–[74].

The factor “Indulgence” describes occasional eating in contexts of pleasure and self-satisfaction. This factor is consistent with a previous study in which a negative association was reported between perceived-pleasure and overconsumption of chocolate amongst women [75]. Neurophysiological data also suggest that it is desire, and not the pleasure of actual consumption, that is associated to overconsumption of food [76].

Lastly, the factor ‘Emotional needs’ indicates that sweetened product consumption may be triggered by occasional negative emotional feelings, either depression-related feelings including sadness, boredom, and loneliness, or stress-related feelings such as anxiety. This factor aligns well with strong support found in previous research. For instance, the ACQ contains a craving factor with items describing negative emotional feelings, such as “When I am down, ‘When I am bored”, and ‘To cheer me up” [27]. Negative mood (as measured by anxiety, fatigue and depression scales) is correlated with craving intensity ratings in individuals who crave sweetened foods [33]. Experimental studies have also indicated an association between stress or negative mood and consumption of savory foods in emotional eaters [77], [78]. Underlying such associations is research indicating that sweet taste may alleviate dysphoric mood or stress through dopaminergic and opioidergic neurotransmission in the brain [79]–[81]. This therapeutic effect may be specific to food and drinks rich in sweet content [82] or to the sweet taste [83]. The factor ‘Emotional needs’ could influence daily consumption of sweetened products in individuals who are in a permanent state of depressed or distressed feelings. Indeed, no association has been reported between emotional eating and consumption of macronutrients [84], [85].

### Implications

This study has important implications for the prevention and management of obesity in the Middle Eastern Canadian community, and in other urban migrant populations. Our findings showed that sweetened products are consumed in various situational contexts and served different purposes. These purposes are linked to the intrinsic characteristics of sweetened products; they are convenient, cheap, energy dense, highly palatable, attractive, and can alleviate negative mood or stress. We also identified an important distinction between the situational contexts associated to the daily and occasional consumption of sweetened products. Future health prevention efforts to reduce sugar intake should focus on this distinction. This is particularly important to address the burden of obesity and chronic diseases in migrant western populations, since they are exposed to socioeconomic and work-related challenges [51], and a new food environment where sweetened products are abundant and heavily advertised [86]. Furthermore, the instrument we developed can be tested and used in future studies to measure the relationships between individual factors, situational contexts, and sweetened food and drink product consumption. Such work will help to understand the complex interactions between behaviour, context and diet.

### Strength and limitations

The strength of this study is the use of a mixed method sequential design and of triangulation to identify and describe the situational contexts of sweetened product consumption. This design enabled the development of a tool with initial validation in regards to face and construct validity, reliability and predictive validity.

This study has some limitations. First, the selection of participants rested on self-selection and therefore introduced a potential volunteer bias. Non-respondents would mainly include those individual not attending church or not participating in activities organized by the church communities. However, the vast majority of Canadian Arabs (94%) described themselves as being part of a religious faith. Also, there are no reasons to believe that characteristics of church attenders and non-attenders differ in terms of the variables examined in this study. The results of our study can therefore be generalized to the Catholic Middle Eastern Canadian community. However, the source community targeted for this research exhibited a restricted range of medium to high socioeconomic status, and thus, our assessment does not account for potential effects of lower income and education on dietary behaviors. Second, the absolute sample size used in the second stage of the study was small and thus statistical power may be regarded as limited. However, necessary sample size is not a matter of absolute size: it depends on multiple aspects including the level of communality of the items, the fit of the data, and the level of over-determination of the factors [60], [61]. Such criteria were used to ensure that data in the exploratory factor analysis were strong and that the factor structure was stable. Lastly, the current study assessed an initial validation of the SCISPC. More work is under way to furthermore validate the psychometrics properties of the SCISPC including its assessment in other populations and other settings.

## Conclusion

The novelty and importance of this study is its use of an exploratory sequential mixed-method design with a rationale of triangulation to identify and describe the situational contexts associated with consumption of sweetened food and drink products in a migrant Canadian community. In order to fulfill the study objective, we developed and assessed an initial validation of the Situational Context Instrument for Sweetened Product Consumption. Overall, the instrument has excellent internal consistency, adequate test-retest reliability, and content and predictive validity. Its psychometric properties warrant further research.

Furthermore, we found that the daily consumption of sweetened foods and drink products can be predicted by unplanned and irregular patterns of eating, this occurring in the contexts of snacking, and energetic demands. The situational contexts in which sweetened products are consumed daily in the western environment may be linked to unique properties of sweetened products, as well as structural characteristics which are typical of the modern food environment [87] and affected by intrinsic features of the global food system [13], [88]. However, before such an ecological perspective can be used to frame our data, both individual and environmental factors, and their interaction, should to be evaluated.
